# BILL-Cadherin/Cadherin-17 Contributes to the Survival of Memory B Cells

**DOI:** 10.1371/journal.pone.0117566

**Published:** 2015-01-22

**Authors:** Shuichi Funakoshi, Takeyuki Shimizu, Osamu Numata, Manabu Ato, Fritz Melchers, Kazuo Ohnishi

**Affiliations:** 1 Graduate School of Life and Environmental Sciences, University of Tsukuba, Ibaraki, Japan; 2 Department of Immunology, Kochi Medical School, Kochi University, Kochi, Japan; 3 Faculty of Life and Environmental Sciences, University of Tsukuba, Ibaraki, Japan; 4 Research Group of Lymphocyte Development, Max Planck Institute for Infection Biology, Berlin, Germany; 5 Department of Immunology, National Institute of Infectious Diseases, Tokyo, Japan; Massachusetts General Hospital, UNITED STATES

## Abstract

Memory B cells (MBCs) and long-lived plasma cells (LLPCs) are responsible for immunological “memory”, which can last for many years. The long-term survival niche for LLPCs in the bone marrow is well characterized; however, the corresponding niche for MBCs is unclear. BILL-cadherin/cadherin-17 (CDH17) is the only member of the cadherin superfamily that is expressed on mouse B lymphocytes in a spatiotemporally regulated manner. Here, we show that half of all MBCs regain expression of CDH17 during the later stage of development. The maintenance of high affinity antigen-specific serum antibodies was impaired in CDH17^-/-^ mice and the number of antigen-specific MBCs was reduced as compared to wild-type mice (WT). Also, specific responses to secondary antigens were ablated in CDH17^-/-^ mice, whereas primary antibody responses were the same as those in WT mice. Cell cycle analysis revealed a decline in the proliferation of CDH17^-^ MBCs as compared to CDH17^+^ MBCs. In addition, we identified a subpopulation of splenic stromal cells, MAdCAM-1^+^ blood endothelial cells (BEC), which was CDH17^+^. Taken together, these results suggest that CDH17 plays a role in the long-term survival of MBCs, presumably via an “MBC niche” comprising, at least in part, BEC in the spleen.

## Introduction

BILL-cadherin/cadherin-17 (CDH17) is a cell adhesion molecule that belongs to the cadherin superfamily, a large group (more than 100 members) of cell adhesion molecules with properties similar to those of integrins and selectins. Cadherins are Ca^2+^-dependent adhesion molecules characterized by their unique extracellular domains, which primarily comprise multiple cadherin-repeats. Cadherins primarily mediate homotypic (cell to cell) adhesion; therefore, they play important roles in intercellular recognition during embryogenesis and morphogenesis [1, 2].

CDH17 contains seven cadherin domains and has no catenin-binding region within its cytoplasmic domain; the latter feature means that CDH17 is classified as a non-classical cadherin [3, 4]. CDH17 requires Ca^2+^ for homotypic adhesion [3, 5]; however, heterotypic adhesion to E-cadherin has been reported [6]. In mice, CDH17 is expressed in the spleen, bone marrow, and intestine [3, 7], whereas in rats it is also expressed in the liver [4].

We previously showed that precursor B cells express CDH17 during early development in the bone marrow [8]. T cells, however, do not express CDH17 [3, 8]. CDH17 is expressed during the pro-B/pre-B-I stages before being downregulated during the pre-B-II stage; it is then upregulated again on immature B cells [3]. CDH17-deficient mice have an increased number of pro-B cells and a reduced number of immature B cells, indicating that CDH17 plays a role(s) in early B cell development (i.e., during transition from the pro/pre-B-I stage to the pre-B-II stage) [8]. Also, the size and the number of germinal centers (GC) in non-immunized CDH17^-/-^ mice is reduced, and the antibody response to a T-independent antigen is decreased as compared to WT mice [8]. These observations suggest that CDH17 might also play a role in late B cell development.

The aim of the present study was to compare T cell-dependent antigen-specific antibody responses to nitrophenylated chicken gammaglobulin (NP-CGG) in wild-type (WT) mice with those in CDH17^-/-^ mice. The results showed that CDH17 contributes to the long-term survival of memory B cells. Furthermore, we identified a population of MAdCAM-1^+^ blood endothelial cells (BEC) that is CDH17^+^. Taken together, these results suggest that CDH17 is involved in the long-term survival of MBCs, and that CDH17^+^ BEC are a candidate for the elusive “MBC niche”. The findings of the present study provide crucial clues that will improve our understanding of the mechanisms underlying long-term MBC survival.

## Materials and Methods

### Mice and ethics statements

CDH17 knock-out mice (BT262) were generated as previously described [8]. The KO mice were backcrossed onto a C57BL/6 background for ten generations. CDH17^+/+^ and CDH17^-/-^ homozygous littermates were used for all experiments. All mice were bred and maintained in a specific-pathogen-free (SPF) facility. All animal experiments were performed according to institutional guidelines and with the approval of the National Institute of Infectious Diseases Animal Care and Use Committee (Permit Number: 213045-2). Mice were housed under a 12 hour light/dark cycle, and provided with food and water *ad libitum*. All efforts were made to minimize suffering. Mice were immunized intraperitoneally with antigen in a volume of less than 200 μL containing 50% Alum adjuvant. Blood samples were drawn from the tail vein and less than 100 μL was collected each time. Mice were euthanized by carbon dioxide inhalation and the spleens were explanted.

### Antibodies and reagents

A rat monoclonal antibody (BD1B) against mouse CDH17 was raised as previously described [3]. The following antibodies and reagents were purchased from BD Pharmingen: PE/Cy7-anti-mouse IgM (catalog number, 552867; working dilution, 1:100), biotin-anti-mouse CD11a/integrin α_L_ (557365; 1:100), biotin-anti-mouse CD18/integrin β_2_ (557439; 1:100), FITC-anti-mouse CD21 (553818; 1:100), FITC-anti-mouse Igλ (553434; 1:100), PE-anti-mouse CD31 (561073; 1:100), PE-anti-mouse CD23 (01235B; 1:50), APC-anti-mouse CD138 (558626; 1:100), PE-anti-mouse CD45R/B220 (01125B; 1:100), and PE-streptavidin (554061; 1:500). The following antibodies were purchased from eBioscience: eFluor 450-anti-mouse IgD (48-5993-80; 1:100), Alexa Fluor 488-anti-mouse/human GL7 (53–5902–80; 1:200), Pacific Blue-anti-mouse/human CD45R/B220 (57-0452-82; 1:100), Alexa Fluor 700-anti-mouse CD38 (56-0381-82; 1:100), and Alexa Fluor 488-anti-mouse Podoplanin (gp38) (53-5381-80; 1:100). The following antibodies were purchased from BioLegend: Brilliant Violet 421 anti-mouse IgG1 (406615; 1:50), PerCP/Cy5.5-anti-mouse IgG1 (406611; 1:100), PE-anti-mouse CD144/VE-cadherin (138009; 1:100), Alexa Fluor 647-anti-mouse CD80 (104717; 1:100), PE/Cy5-anti-mouse Gr-1 (108410; 1:100), PE/Cy5-anti-mouse TER-119 (116210; 1:100), PE/Cy5-anti-mouse CD3ε (100310; 1:100), APC/Cy7-anti-mouse/human CD45R/B220 (103224; 1:100), APC/Cy7-anti-mouse CD45 (103116; 1:100), FITC-anti-mouse IgG1 (406606; 1:100), biotin-anti-mouse MAdCAM-1 (120705; 1:100), Alexa Fluor 488-anti-mouse MAdCAM-1 (120707; 1:50), Alexa Fluor 700-anti-human CD19 (302225; 1:100), and FITC-anti-human CD27 (302805; 1:100). Alexa Fluor 647-anti-mouse CD169 (MCA947A647; 1:100) was purchased from AbD Serotec. Pacific Blue-anti-BrdU (B35129; 1:100), Qdot 655-streptavidin (Q10123MP; 1:100), Qdot 705-streptavidin (Q10163MP; 1:100), and O-phenylenediamine (002003) were purchased from Invitrogen. Alexa Fluor 488-anti-mouse CXCR3 (FAB1685G; 1:100) and Alexa Fluor 488-anti-mouse CCR6 (FAB590G; 1:100) were purchased from R&D Systems. The following antibody was obtained from Abcam: FITC-anti-mouse CD273 (ab59872; 1:100). Biotin-anti-mouse IgD (1120–08; 1:100), HRP-anti-mouse IgM (1020-05; 1:5000), and HRP-anti-mouse IgG1 (1144–05; 1:50000) were obtained from Southern Biotech. Alexa Fluor 430-streptavidin (S11237; 1:100) was obtained from Molecular Probes. The following reagents were purchased from Sigma-Aldrich: 7-AAD (A9400-1MG; 10 μg/mL) and 5-bromo-2′-deoxyuridine (BrdU; B5002-250MG). The following reagents were obtained from Biosearch Technologies: (4-hydroxy-3-nitro-phenyl)-acetyl (NP)_48_-CGG, NP_4_-BSA, and NP_16_-BSA. Dispase (17105-041; 0.8 mg/mL) and collagenase IV (17104–019; 50 U/mL) were from Gibco. PE/Texas Red-streptavidin (IM3326, 1:100) was from Beckman Coulter. Tissue-Tek O.C.T. Compound (4583) was from Sakura. Finally, the following antibodies and reagents were generated in-house: biotin-BD1B, Alexa Fluor 647-BD1B, PE/Texas Red-BD1B, anti-mouse CD16-2 (2.4G2, 20 μg/mL), and NP_44_-CGG. PE-(4-hydroxy-5-iodo-3-nitro-phenyl)-acetyl (NIP)_25_ was a kind gift from Dr. Takahashi (Department of Immunology, National Institute of Infectious Diseases, Tokyo, Japan). The closely-related epitopes, NP and NIP, react with the same spectrum of specific antibodies [9]. NIP is widely used for flow cytometry because it has high affinity for NP-specific B cells. Therefore, NP was used in the ELISA because the number of NP-haptens on the CGG-carrier had to be controlled. NIP was used for flow cytometry.

### Immunizations

For the primary antibody response experiments, 3–4-month-old CDH17^+/+^ and CDH17^-/-^ littermates were immunized intraperitoneally with 50 μg of NP_48_-CGG precipitated with 100 μL of Imject Alum adjuvant (Thermo Scientific, 77161).

For the secondary antibody response experiments, 10–11-week-old CDH17^+/+^ and CDH17^-/-^ littermates were immunized intraperitoneally with 5 μg of NP_44_-CGG precipitated with 100 μL of Imject Alum adjuvant. The mice were then boosted with an intraperitoneal injection of NP_44_-CGG (2.5 μg) 50 weeks later.

### ELISA

Flat-bottomed 96-well plates (Immuno-MaxiSorp; Nunc 442404) were coated with 5 μg/mL of NP_1.6_-BSA (to detect high affinity anti-NP antibodies) or with 5 μg/mL NP_4_-BSA or NP_16_-BSA (to detect total affinity anti-NP antibodies) and incubated at room temperature for 2 h or at 4°C overnight, followed by incubation with 1% BSA in PBS/0.05% Tween 20 (Sigma, P-1379). Sera collected from immunized littermates were serially diluted, added to the plates, and incubated for 1 h at room temperature, followed by incubation with HRP-anti-mouse IgM and HRP-anti-mouse IgG1 antibodies. Hydrogen peroxide in citrate buffer and O-phenylenediamine were used as the chromogen. Optical density was measured at 490 nm. NP-specific IgM and IgG1 monoclonal antibodies (established in-house) were used as standards to calculate the relative antibody titers and affinities.

### Flow cytometry

Mice were euthanized by CO_2_ inhalation. Spleens were excised and spleen cell suspensions prepared by mechanical disruption in staining buffer (Hank’s Balanced Salt Solution (HBSS) containing 1% bovine serum albumin and 0.05% sodium azide), followed by filtering through a stainless steel mesh. To detect T1, T2, marginal zone (MZ), and mature B cells, cell suspensions were prepared from spleens isolated from CDH17^+/+^ and CDH17^-/-^ mice. Briefly, spleens were treated with 2.4G2 (20 μg/mL) for 30 min on ice to block Fcγ receptors and then stained with PE/Cy5-anti-Gr-1, PE/Cy5-anti-Ter-119, PE/Cy5-anti-CD3ε, APC/Cy7-anti-B220, eFluor 450-anti-mouse IgD, PE/Cy7-anti-mouse IgM, and Alexa Fluor 647-BD1B for 60 min on ice (working dilutions are provided in the antibodies and reagents section). T1, T2, and MZ B cells were also stained with PE-anti-CD23 and FITC-anti-CD21. After washing well, the cells (5 × 10^6^ cells/mL) were suspended in staining buffer containing 5 μg/mL propidium iodide (PI). About 1 × 10^6^ cells were analyzed in a BD FACSAria III (BD Biosciences) and dead cells (PI^+^) were excluded. This was the basic protocol for flow cytometry analysis and was used for all analyses described below.

To detect NP-specific GC B cells, cell suspensions were prepared from spleens on Day 12 post-immunization with NP-CGG, treated with 2.4G2 to block Fcγ receptors, and then stained with PE/Cy5-anti-Gr-1, PE/Cy5-anti-Ter-119, PE/Cy5-anti-CD3ε, APC/Cy7-anti-B220, eFluor 450-anti-mouse IgD, PE/Cy7-anti-mouse IgM, Alexa Fluor 700-anti-CD38, PE-NIP_25_, Alexa Fluor 488-anti-GL7, and PE/Texas Red-BD1B. PI was added to exclude PI^+^ dead cells.

To detect NP-specific plasma cells (PCs), littermates were immunized with NP-CGG, followed by a boost immunization on Day 196. Cell suspensions were isolated from spleens on Day 7 after the boost immunization, treated with 2.4G2 to block Fcγ receptors, and then stained with PE/Cy5-anti-Gr-1, PE/Cy5-anti-Ter-119, PE/Cy5-anti-CD3ε, Pacific Blue-anti-B220, APC-anti-CD138, PE-NIP_25_, FITC-anti-Igλ biotin-BD1B, and Alexa Fluor 430-streptavidin. PI was added to exclude PI^+^ dead cells.

To detect NP-specific MBCs, cell suspensions were isolated from the spleens of littermates immunized with NP-CGG, treated with 2.4G2 to block Fcγ receptors, and then stained with PE/Cy5-anti-Gr-1, PE/Cy5-anti-Ter-119, PE/Cy5-anti-CD3ε, APC/Cy7-anti-B220, eFluor 450-anti-mouse IgD, PE/Cy7-anti-mouse IgM, Alexa Fluor 700-anti-CD38, PE-NIP_25_, FITC-anti-mouse IgG1, and Alexa Fluor 647-BD1B. PI was added to exclude PI^+^ dead cells.

To detect surface markers on MBCs, cell suspensions were isolated from the spleens of littermates, treated with 2.4G2 to block Fcγ receptors, and then stained with PE/Cy5-anti-Gr-1, PE/Cy5-anti-Ter-119, PE/Cy5-anti-CD3ε, APC/Cy7-anti-B220, eFluor 450-anti-mouse IgD, PE/Cy7-anti-mouse IgM, Alexa Fluor 700-anti-CD38, PerCP/Cy5.5-anti-mouse IgG1, Qdot 705-streptavidin, Alexa Fluor 647-BD1B, Alexa Fluor 488-anti-mouse CXCR3, biotin-anti-mouse CD18, Alexa Fluor 488-anti-mouse CCR6, biotin-anti-mouse CD11a, biotin-BD1B, Alexa Fluor 647-anti-mouse CD80, FITC-anti-mouse CD273, and PE-anti-mouse CD144. PI was added to exclude PI^+^ dead cells.

### Cell cycle analysis

To examine the cell cycle status of IgG1^+^ MBCs, CDH17^+/+^ and CDH17^-/-^ littermates (17 ± 3-months-old) received an intraperitoneal injection of BrdU (1 mg) 1 h before sacrifice. Cell suspensions were isolated from the spleens, treated with 2.4G2 to block Fcγ receptors, and then stained with APC/Cy7-anti-B220, PE/Cy7-anti-mouse IgM, Alexa Fluor 700-anti-CD38, PE-NIP_25_, FITC-anti-mouse IgG1, Alexa Fluor 647-BD1B, biotin-anti-mouse IgD, and PE/Texas Red-streptavidin. The cells were fixed with 1% paraformaldehyde/PBS at 0°C for 1 hr and permeabilized with 0.05% NP-40/1% paraformaldehyde/PBS at 4°C overnight. Cells were then washed twice with 1% glycine/PBS and treated with DNase I solution (1 mg/mL DNase I, 1 mM CaCl_2_, and 1 mM MgCl_2_ in PBS) at 37°C for 10 minutes, followed by staining with Pacific Blue-anti-BrdU at room temperature for 45 minutes. Cells were suspended in 7AAD and the amount of DNA was measured by flow cytometry.

### Stromal cell analysis

To detect CDH17^+^ stromal cells, spleen cells were fractionated according to the method of Fletcher et al. [10]. Briefly, spleens were pierced and treated with a dispase (0.8 mg/mL)/collagenase IV (50 U/mL) enzyme mixture at 37°C for 20 minutes. The cell suspensions were generated by passage through a stainless sieve, then treated with 2.4G2 to block Fcγ receptors and stained with APC/Cy7-anti-mouse CD45, PE/Cy5-anti-Ter-119, Alexa Fluor 488-anti-mouse Podoplanin (gp38), PE-anti-mouse CD31, Alexa Fluor 647-BD1B, biotin-anti-mouse MAdCAM-1, and Qdot 705-streptavidin. PI was added to exclude PI^+^ dead cells. Stained cells were then analyzed by flow cytometry as described above.

### Confocal microscopy

Frozen spleen sections (6 μm thick) were treated with the Catalyzed Signal Amplification (CSA) System (Dako, K1500) to block endogenous peroxidase activity, followed by treatment with the Endogenous Biotin-Blocking Kit (Invitrogen, E21390) to block endogenous biotin and 2.4G2 to block Fcγ receptors. Sections were then stained with biotin-BD1B. After washing, the tissue sections were treated with a streptavidin biotin complex (CSA system) followed by the Individual Indirect Tyramide Reagent (PerkinElmer, SAT700001EA) to amplify the biotin-BD1B signal. Sections were then stained with Qdot 655-streptavidin, Alexa Fluor 647-anti-mouse CD169, Brilliant Violet 421 anti-mouse IgG1, and Alexa Fluor 488-anti-mouse MAdCAM-1. Immunostained tissue sections were visualized under a Zeiss LSM710 confocal microscope. Data were analyzed using ZEN software.

### Statistical analysis

Microsoft Excel was used for statistical analysis. The error bars on the figures represent the standard deviation from the mean. Serum antibody titers, the differential between the pre-boost and post-boost titers, the percentage of MBCs, and the MBC cell cycle indices were tested using the Mann-Whitney U-test. All other data were tested using Student’s t-test. In all cases, differences were considered significant at p<0.05 (*p<0.05, **p<0.01, and ***p<0.001).

## Results

### Differentiation-dependent regulation of CDH17 expression during late stages of B cell development

**Figure 1:**
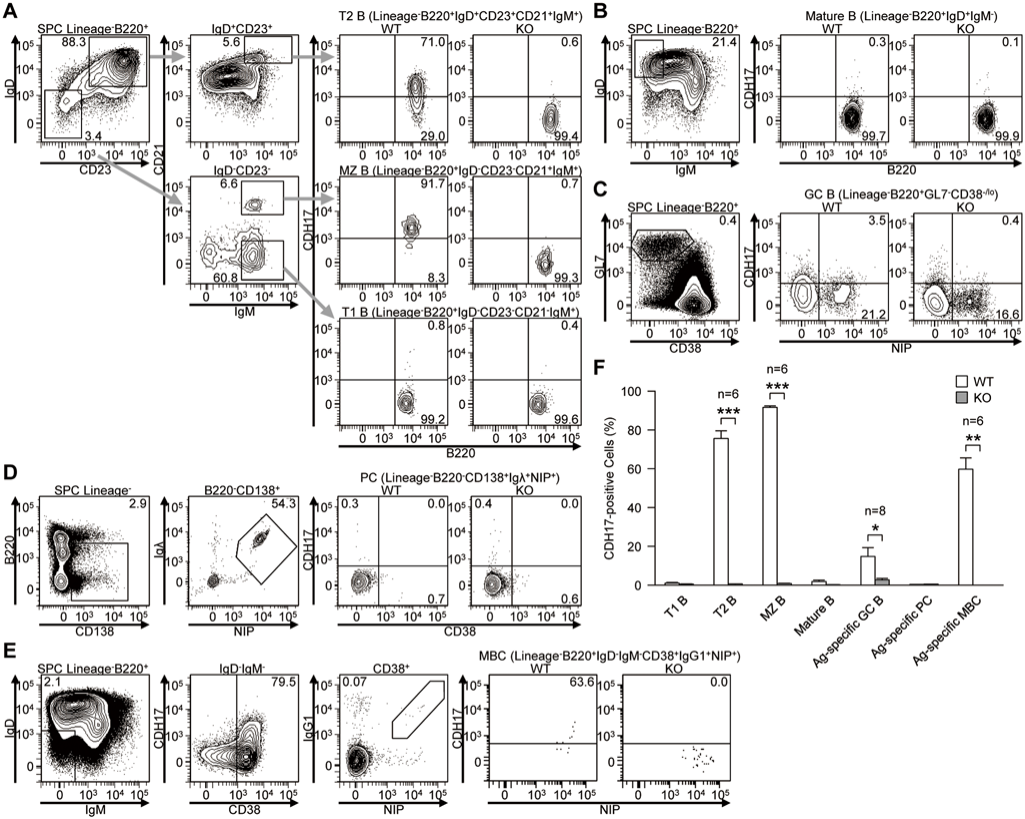
The expression of CDH17 is regulated differentiation-dependently during B cell development in spleen. (A–E) Expression of CDH17 on spleen cells and bone marrow cells isolated from wild-type (WT) and CDH17^-/-^ (KO) mice were analyzed by flow cytometry: (A) T1 B cells (Lin^-^B220^+^IgD^-^CD23^-^CD21^-^IgM^+^), T2 B cells (Lin^-^B220^+^IgD^+^CD23^+^CD21^+^IgM^+^), and MZ B cells (Lin^-^B220^+^IgD^-^CD23^-^CD21^+^IgM^+^); (B) Mature B cells (Lin^-^B220^+^IgD^+^IgM^-^); (C) Antigen-specific GC B cells (Lin-B220^+^GL7^+^CD38^low/-^NIP^+^); (D) Antigen-specific PCs (Lin^-^B220^-^CD138^+^Igλ^+^NIP^+^); (E) Antigen-specific MBCs (Lin^-^B220^+^IgD^-^IgM^-^CD38^+^IgG1^+^NIP^+^). Numbers adjacent to the gates indicate the percentage (%) of cells in the respective parental gates. (F) The percentages of CDH17^+^ cells within various B cell populations are plotted on a bar graph. The percentage of CDH17^+^ cells relative to the total number of cells in the parental gates (indicated on the top of the corresponding flow cytometric contour plot) was calculated. In (C), the percentage of antigen-specific cells within the parental gates was calculated (n = 2 (PCs), n = 8 (GC B), n = 6 (others); *P≤0.05, **P≤0.01, ***P≤0.001 (Student’s t-test)).

To investigate the possible roles of CDH17 in late B cell responses, we first analyzed the expression of CDH17 by various splenic B cell populations in WT immunized with NP-CGG and in KO mice. Whereas transitional-1 B cells (T1 B; Lin^-^B220^+^IgD^-^CD23^-^CD21^-^IgM^+^) were CDH17^-^, almost all transitional-2 B cells (T2 B; Lin^-^B220^+^IgD^+^CD23^+^CD21^+^IgM^+^) were CDH17^+^. Marginal zone B cells (MZ B; Lin^-^B220^+^IgD^-^CD23^-^CD21^+^IgM^+^) were also CDH17^+^ (Fig. 1A). Both mature B cells (Lin^-^B220^+^IgD^+^IgM^-^) and antigen-specific PCs (Lin^-^B220^-^CD138^+^IgG^+^NIP^+^) were CDH17^-^ (Fig. 1B, D). A small fraction of antigen-specific GC B cells (GC B; Lin^-^B220^+^GL7^+^CD38^lo/-^NIP^+^) were CDH17^+low^ (Fig. 1C). Interestingly, about half of antigen-specific MBCs (Lin^-^B220^+^IgD^-^IgM^-^CD38^+^IgG1^+^NIP^+^) were CDH17^+^ (Fig. 1E). These data are consistent with DNA microarray data published in a previous report showing specific CDH17 expression by MBCs [11]. To further characterize the CDH17^+^ and CDH17^-^ MBCs, we next examined the expression of chemokine/cytokine receptors and other adhesion molecules (CXCR3, CCR6, integrin-β2, integrin-αL, VE-cadherin, CD80, and CD273) (S1 Fig.). Among these molecules, integrin-α_L_, integrin-β_2_, and CD273 were expressed at slightly higher levels in CDH17^+^ MBCs than in CDH17^-^ MBCs.

These results show that the expression of CDH17 is tightly regulated during the late stages of B cell development: it is downregulated in T1 B cells, upregulated in T2 B cells, downregulated in mature B cells, and regained in MBCs during the GC reaction (Fig. 1F). These observations raise the possibility that BILL-cadherin might be involved in antibody affinity maturation and/or B cell memory.

### The maintenance of serum antibody affinity is impaired in CDH17^-/-^ mice

**Figure 2:**
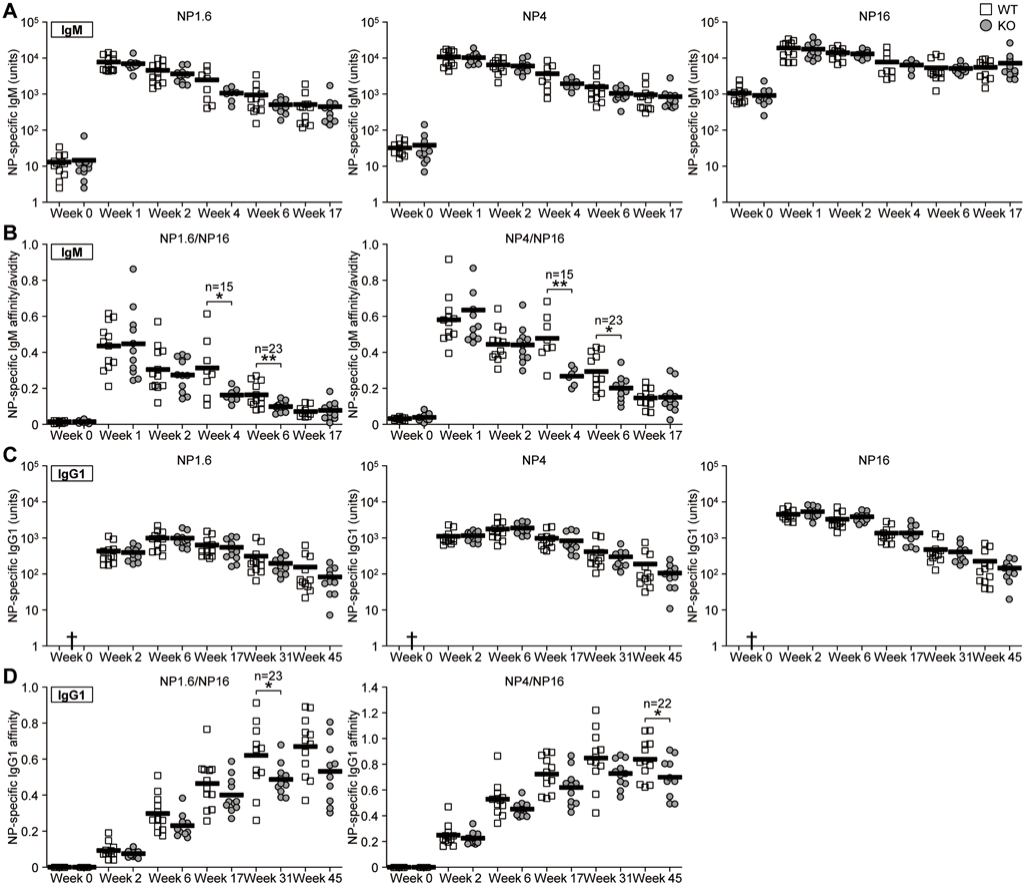
High affinity NP-specific antibodies decay more rapidly in the sera of CDH17^-/-^ mice. CDH17^-/-^ mice and their WT littermates were immunized with NP-CGG in alum. (A, C) The titers of serum IgM (A) and IgG1 (C) antibodies against NP-BSA were measured in an ELISA. Antibody titers are expressed in terms of units derived from a panel of reference monoclonal NP-specific antibodies. Each symbol represents an individual mouse. The mean values are represented by the bold lines. The titers of high affinity (NP1.6), medium affinity (NP4), and total affinity (NP16) antibodies are shown. (B, D) Affinity ratios of IgM (B) or IgG1 (D) were calculated for high affinity (NP_1.6_/NP_16_) and medium affinity (NP_4_/NP_16_) antibodies using the data in (A) and (C). Statistical significance was tested using Student’s t-test (n = 15 (Week 4); n = 23 (other times); *P≤0.05, **P≤0.01). †Lower than 1 unit.

To elucidate the role of CDH17 in affinity maturation and MBC formation, we compared the antibody responses in WT and KO mice. Mice were immunized with NP-CGG, and antigen-specific serum IgM (Fig. 2A) and IgG1 (Fig. 2C) antibody titers were measured. Antibody affinity for NP_1.6_, NP_4_, and NP_16_-BSA was examined in an ELISA. There were no significant differences in the total NP-specific serum IgM and IgG1 titers between WT and KO mice at 45 weeks after the primary immunization. However, when we examined WT and CDH17^-/-^ mice for the presence of high affinity NP-specific serum antibodies, we found that the affinity of NP-specific serum IgM was lower in CDH17^-/-^ mice than in WT mice at 4–6 weeks post-immunization (Fig. 2B), and that the affinity of IgG1 was lower in CDH17^-/-^ mice from week 31 post-immunization (Fig. 2D). Because there were no significant differences in the anti-NP_16_ total antibody titer between WT and CDH17^-/-^ mice, the lower affinity of IgM and IgG1 in CDH17^-/-^ mice must be due to a reduction in the high affinity anti-NP_1.6_ and anti-NP_4_ antibody titers. Since no significant differences were detected between WT and CDH17^-/-^ mice during the early stages of immunization, the results also suggest that CDH17 becomes important during the later stages of an immune response, e.g., by promoting long-term maintenance of high affinity antigen-specific antibody titers.

### The secondary antibody response is markedly impaired in CDH17^-/-^ mice

**Figure 3:**
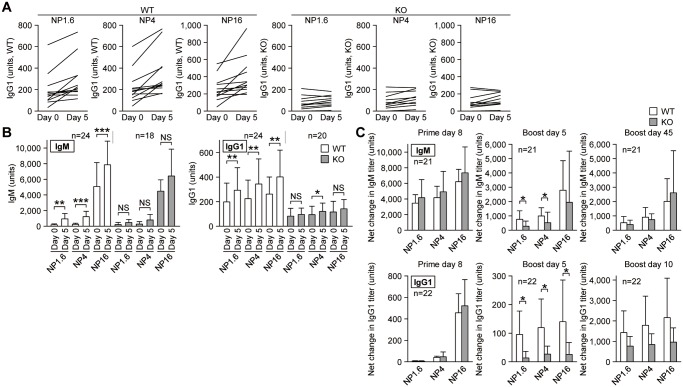
The secondary NP-specific antibody response is impaired in CDH17^-/-^ mice. CDH17^-/-^ mice and their WT littermates received a primary immunization with NP-CGG in alum. Mice were boost immunized (without alum) 50 weeks later. Serum titers of anti-NP IgM and IgG1 antibodies were measured in an ELISA. (A) The changes in NP-specific serum IgG1 titer in individual mice are plotted on a line graph (titers are expressed as described in the legend to Fig. 2). The horizontal axis shows the day of blood sampling (Day 0 = the day of boost; Day 5 = 5 days post-boost). Each line represents an individual mouse. (B) The average titers of NP-specific serum IgM (left) and IgG1 (right) calculated from the data shown in (A) are plotted on a bar graph. Statistical significance was calculated using a paired Student’s t-test (n = 24 (WT, IgM); n = 18 (KO, IgM); n = 24 (WT, IgG1); n = 20 (KO, IgG1)). (C) The net increment of NP-specific serum IgM titers (top) and IgG1 titers (bottom) after the primary immunization (left) and boost immunization (middle, 5 days after the boost; right, 45 days after the boost) was calculated by subtracting the pre-immune (left) or pre-boost (middle and right) serum titers (n = 24 (WT, IgM); n = 24 (WT, IgG1); n = 18 (KO, IgM); n = 20 (KO, gG1)). NS, not significant (P>0.05). *P≤0.05, **P≤0.01, ***P ≤0.001.

High affinity antibody titers are thought to be maintained by MBCs and LLPCs. Because we found PCs to be CDH17^-^ and about half of MBCs to be CDH17^+^ (Fig. 1), one possible explanation for the loss of high affinity antibodies could be impaired maintenance of high affinity-NP-specific MBCs in CDH17^-/-^ mice. To test this hypothesis, we examined secondary antibody responses to NP-CGG. WT and CDH17^-/-^ mice were boost immunized with NP-CGG in the absence of alum adjuvant at around 50 weeks post-priming. The increases in NP-specific serum antibody titers at Day 5 post-boost were then compared with those before the boost. We found that the NP-specific serum IgM and IgG1 titers in WT mice increased markedly at Day 5 post-boosting. On the other hand, there was only a small increase in boosted CDH17^-/-^ mice (Fig. 3). The difference was most evident when changes in antibody titers were compared between individual mice. Antibody titers increased significantly in WT mice, whereas only a small increase, or even a decrease, was observed in individual CDH17^-/-^ mice (Fig. 3A).

Because the NP-specific IgG1 titers at the time of the boost immunization were different between WT and CDH17^-/-^ mice, we next compared the net difference in NP-specific serum IgG1 antibody titers before and after the secondary immunization (Fig. 3B). The differences in NP-specific serum IgM (excluding total NP_16_-specific antibodies) and IgG1 titers were significantly higher in WT mice 5 days after the secondary immunization, but not in CDH17^-/-^ mice. This bias was more evident for IgG1 antibodies (Fig. 3C). Because there were no significant differences in the NP-specific serum antibody titer between WT and CDH17^-/-^ mice after the primary immunization, it appears that the defect in CDH17^-/-^ mice is specific for the secondary antibody response.

In conclusion, these results showed that the secondary antibody response is functional, but impaired, in CDH17^-/-^ mice, indicating that CDH17 affects the production of antigen-specific antibodies during the secondary immune response.

### CDH17 contributes to the long-term maintenance of MBCs

**Figure 4:**
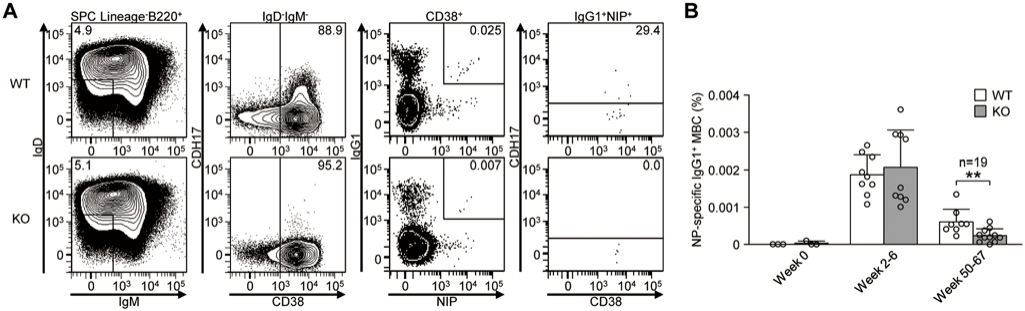
Long-term maintenance of antigen-specific MBCs is impaired in CDH17^-/-^ mice. (A) Antigen-specific IgG1^+^ MBCs (Lin^-^B220^+^IgD^-^IgM^-^CD38^+^IgG1^+^NIP^+^) obtained from CDH17^-/-^ mice and their WT littermates at 52 weeks after primary immunization with NP-CGG in alum were analyzed by flow cytometry. Numbers represent the percentage (%) of the indicated cell populations in the respective parental gates (shown on top of the panels). (B) The percentages of antigen-specific IgG1^+^ MBCs in their respective B220^+^ parental gate are plotted on a bar graph. The number of weeks post-immunization are shown for each bin. Each symbol represents an individual mouse (n = 6 (Week 0); n = 18 (Weeks 2–6); n = 19 (Weeks 50–67); **P≤0.01 (Mann-Whitney U-test)).

Because CDH17^-/-^ mice show an impaired secondary antibody response, we next examined whether antigen-specific MBCs were less common in CDH17^-/-^ mice than in WT mice. WT and CDH17^-/-^ mice were immunized with NP-CGG and changes in the percentage of NP-specific IgG1^+^ MBCs (Lin^-^B220^+^IgD^-^IgM^-^CD38^+^IgG1^+^NIP^+^) were monitored by flow cytometry over a long time period (Fig. 4). There was no significant difference in the percentage of NP-specific MBCs between WT and CDH17^-/-^ mice during the first 2–6 weeks post-immunization (Fig. 4B), indicating that the initial rate of MBC development is not affected by CDH17 deficiency. Moreover, the number of progenitor cells, including mature B cells, T1 cells, T2 cells, antigen-specific GC B cells, and the total number of IgG1^+^ MBCs, was almost the same in WT and CDH17^-/-^ mice (S2 and S3 Figs.). However, the number of NP-specific MBCs in CDH17^-/-^ mice was significantly reduced (to about one-third of that in WT mice) after longer periods of time (50–67 weeks) post-immunization (Fig. 4B). These results indicate that CDH17 plays an important role in the long-term maintenance of antigen-specific MBCs, which becomes clear after 50 weeks post-antigen exposure.

### The “*in vivo*” proliferation of CDH17^-^ IgG1^+^ MBCs is significantly retarded

**Figure 5:**
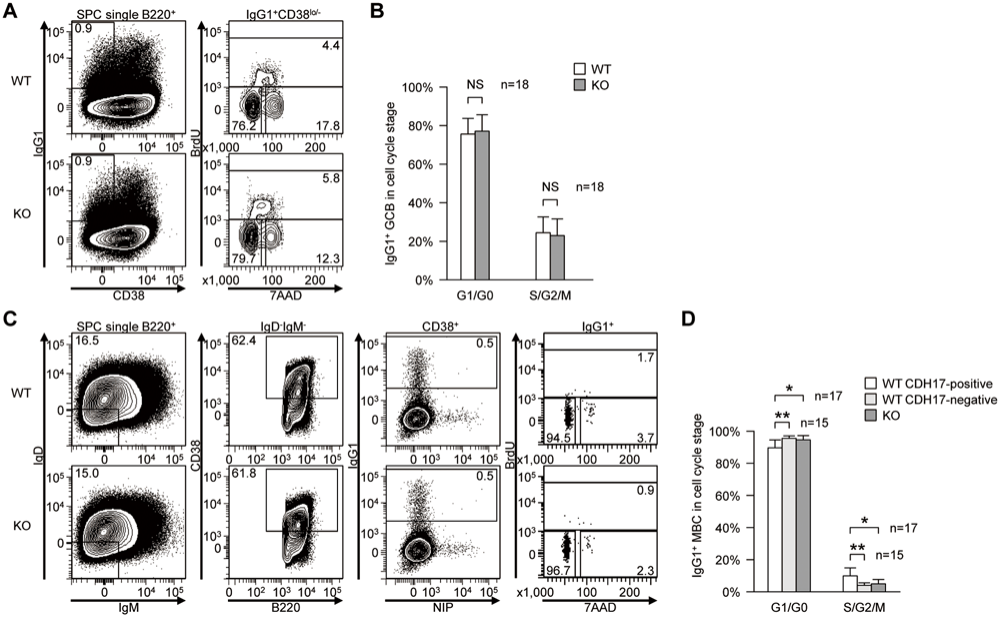
CDH17^+^ switched IgG1^+^ MBCs show increased cell cycle progression. (A) Cell cycle of IgG1^+^ GCB cells (B220^+^IgG1^+^CD38^lo/-^) from CDH17^-/-^ mice and their WT littermates were analyzed by flow cytometry. Numbers indicate the percentage (%) of cells in their respective cell cycle gates (lower left, G0/G1 phase; top, S phase; lower right, G2/M phase). (B) The percentages of the GCB cell population in each indicated cell cycle stage are plotted on a bar graph (n = 18; NS, not significant (P>0.05; Mann-Whitney U-test)). (C) Cell cycle of switched IgG1^+^ MBCs (B220^+^IgD^-^IgM^-^CD38^+^IgG1^+^) from CDH17^-/-^ mice and WT littermates were analyzed by flow cytometry. The numbers indicate the percentage of cells in the respective cell cycle gates. (D) The percentages of switched IgG1^+^ MBCs at the indicated cell cycle stage are plotted on a bar graph (n = 15–17; NS, not significant (P>0.05). *P≤0.05, **P≤0.01 (Mann-Whitney U-test).

To elucidate the role of CDH17 in the long-term maintenance of MBCs, we next examined the influence of CDH17-deficiency on the rate of cell cycle entry, i.e., cell proliferation. Because the number of antigen-specific IgG1^+^ MBCs was very small at 50 weeks post-primary immunization, we performed cell cycle analyses to examine switched IgG1^+^ MBCs rather than antigen-specific IgG1^+^ MBCs. WT and CDH17^-/-^ mice (17 ± 3-months-old) were injected intraperitoneally with BrdU and sacrificed 1 hour later. The cell cycle status of switched IgG1^+^ MBCs (B220^+^IgD^-^IgM^-^CD38^+^IgG1^+^) was then examined by flow cytometry (Fig. 5C). We analyzed the cell cycle status of switched IgG1^+^ MBCs in CDH17^+^ and CDH17^-^ populations separately, and found that CDH17^-^ IgG1^+^ MBCs from WT mice showed the same cell cycle distribution as those from CDH17^-/-^ mice. On the other hand, the cell cycle progression of CDH17^+^ switched IgG1^+^ MBCs was higher in WT mice (Fig. 5D). These results suggest that CDH17 plays a role in the long-term maintenance of MBCs by increasing the rate at which MBCs enter the cell cycle.

### A subpopulation of spleen BEC are CDH17^+^

**Figure 6:**
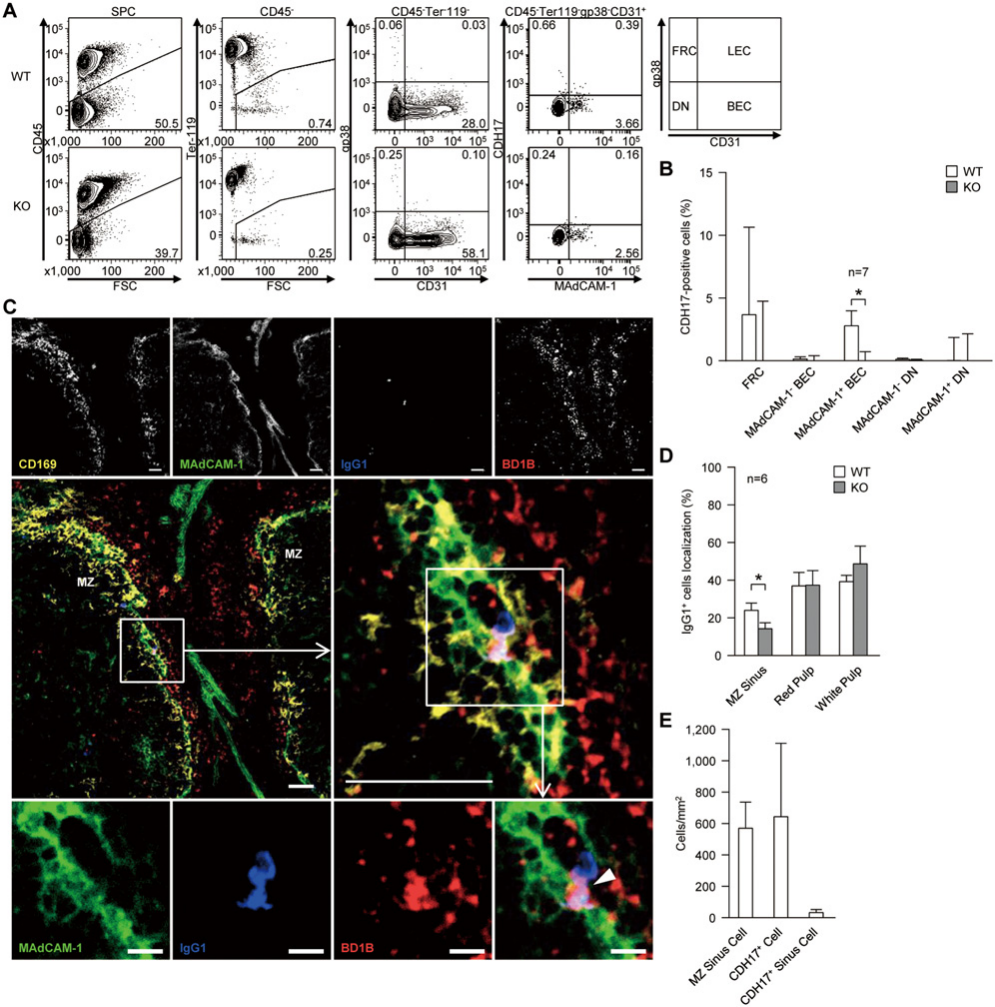
A fraction of BEC is CDH17^+^. (A) Spleens were treated with dispase and collagenase IV to obtain single stromal cells, and cell surface markers were analyzed by flow cytometry. Stromal cells were separated into four subpopulations: fibroblastic reticular cells (FRCs, gp38^+^CD31^-^), lymphatic endothelial cells (LEC, gp38^+^CD31^+^), BEC (gp38^-^CD31^+^), and double-negative (DN) cells (far right panel). Each fraction was then tested for CDH17 expression. The numbers adjacent to the gates indicate the percentage (%) of the indicated cells within their respective parental gates (shown on top of each panel). (B) The percentages of CDH17^+^ stromal cells within the respective parental gates are plotted on a bar graph. The percentages were calculated by subtracting the values of KO mice from those of WT mice (n = 7 (MAdCAM-1^+^ BEC); n = 8 (others)). *P≤0.05 (Student’s t-test). (C) WT mouse spleen was stained with anti-CD169 (yellow), anti-MAdCAM-1 (green), anti-mouse IgG1 (blue), anti-CDH17 (red, BD1B), and analyzed by confocal microscopy. The middle-right micrograph is an enlarged view of the boxed area shown in the middle-left micrograph. The bottom row of images shows each separate color channel and an expanded view of the merged image shown within the box in the middle-right panel. The white arrowhead shown in the bottom right image indicates an IgG1^+^ cell that is adjacent to a CDH17^+^ cell in the MZ sinus. Data are representative of three independent experiments. Scale bars, 50 μm (top and middle row of images) or 10 μm (bottom row). (D) The localization of IgG1^+^ cells in the MZ, red pulp, and white pulp in WT and KO mice are plotted on a bar graph. Values are expressed as the percentage of IgG1^+^ cells localized within each sub-region of the spleen (n = 6; *P≤0.05 (Student’s t-test)). (E) The number of MZ sinus (MAdCAM-1^+^) cells, CDH17^+^ cells, and CDH17^+^ MZ sinus cells (Cdh17^+^MAdCAM-1^+^) in the spleen of WT mice are plotted on a bar graph. Cell numbers were counted in six histological sections of WT spleen. The mean and standard deviation are plotted.

Cells that are retained for long periods of time, such as hematopoietic stem cells, require a specialized niche for survival [12, 13]. MBCs also require such a survival niche to support their long-term maintenance; however, no such niche has been identified. In general, it is thought that appropriate adhesion molecules are required for cells to reside within a specific niche. Therefore, we hypothesized that CDH17 holds MBCs within a specific survival niche via homotypic adhesion. We next examined CDH17^+^ stromal cells as a possible constituent of the MBC survival niche. Mouse spleens were treated with dispase and collagenase IV, and the expression of CDH17 by various stromal cells was examined by flow cytometry (Fig. 6A). We found that a fraction of MAdCAM-1^+^ BEC expressed significant levels of CDH17 (Fig. 6B). However, we noticed that the endothelial cell surface was damaged by dispase/collagenase IV treatment. Although this may cause relatively high background signals, resulting in a lower signal intensity for CDH17, we found that BEC expressed statistically significant levels of CDH17. In this context, we found that fibroblastic reticular cells (FRCs) were also CDH17^+^; however, in this case, the level of specific staining was not statistically significant due to the large standard deviation (Fig. 6B). Thus, FRCs may be vulnerable to the harsh conditions caused by dispase/collagenase IV. Taken together, these results suggest that a CDH17-expressing subpopulation of MAdCAM-1^+^ BEC is a candidate constituent of the MBC survival niche.

In the spleen, MAdCAM-1 is expressed on cells lining the marginal sinuses, which partly overlap with the MOMA-1^+^ marginal zone [14]. CDH17-expressing cells were localized in the region overlapping these MOMA-1^+^ cells and MAdCAM-1^+^ cells; also, MAdCAM-1^+^CDH17^+^ cells were present in this region (although they were infrequent) (Fig. 6C). Overall, about 6% of cells in the MZ sinus (MAdCAM-1^+^) were MAdCAM-1^+^Cdh17^+^ BECs (Fig. 6E). Class-switched IgG1^+^ B cells (B220^+^IgM^-^IgD^-^IgG1^+^) comprise about 0.01% of the splenic B220^+^ B cell population, and about 70% of IgG1^+^ B cells show a CD38^+^ MBC phenotype (B220^+^IgG1^+^IgM^-^IgD^-^CD38^+^). It is not easy to identify MBCs in the spleen by histology; however, small numbers of IgG1^+^ B cells were consistently observed in association with white pulp, red pulp, or the MZ. Indeed, the number of IgG1^+^ B cells found in the MZ was associated with cells those expressing MAdCAM-1 and CDH17 (Fig. 6C). The memory-like IgG1^+^ B cells associated with MAdCAM-1^+^CDH17^+^ BEC were CDH17^+^ (Fig. 6C, bottom panel), suggesting that CDH17 mediates adhesion. Other memory-like IgG1^+^ B cells found on histological examination were also CDH17^+^. The number of IgG1^+^ cells associated with the MZ area was significantly lower in KO mice, whereas IgG1^+^ cells on the red pulp or white pulp were not affected by CDH17-deficiency (Fig. 6D). These observations further support the idea that a CDH17-expressing subpopulation of MAdCAM-1^+^ BEC located near marginal sinus constitutes a possible “MBC niche” in the spleen.

## Discussion

We showed that almost half of MBCs express CDH17. We examined the expression of chemokine/cytokine receptors and other adhesion molecules (which are reported to be expressed on MBCs) on CDH17^+^ and CDH17^-^ MBCs [11, 15]: CXCR3, CCR6, integrin-β2, integrin-αL, VE-cadherin, CD80, and CD273 (S1 Fig.). Of these, integrin-αL, integrin-β2, and CD273 were expressed at slightly higher levels on CDH17^+^ MBCs than on CDH17^-^ MBCs. Integrin-α_L_β_2_ (LFA-1) is involved in B cell localization to the MZ [16], suggesting that CDH17^+^ MBCs are more likely to localize in the MZ than CDH17^-^ MBCs. Also, MBCs are classified into five subsets according to their differential expression of CD273, CD73, and CD80; CD80^hi^ and/or CD273^hi^ cells are considered to be more “memory-like” [15]. The data presented herein suggest that CD273^hi^CDH17^+^ MBCs are more memory-like than CDH17^-^ MBCs. [17]

CDH17-deficient mice showed a reduction in the titer of high affinity anti-NP antibodies over time, without any obvious difference in the total antibody titer. Fig. 2C shows that overall anti-NP16 IgG1 antibody titers changed: titers were almost equal in WT and KO from Week 2 to Week 31, but were slightly lower in KO mice at week 45; however, the difference was not statistically significant. The titers of high affinity IgG (NP1.6 and NP4) antibodies also changed: they were almost equal in WT and KO mice at Weeks 2–6, but were slightly lower in KO mice at Weeks 17–45. One possible explanation for this is that CDH17 is involved in the maintenance of MBCs but not in the formation of MBCs or PCs. We found that during the early phase of the antibody response (Weeks 2–6), there was no difference in antibody titers between WT and KO because PCs developed equally well in both. During the later phase (Weeks 17–45), high affinity antibodies produced by PCs would be replenished; however, these antibodies would be different from those produced by maintained high affinity MBCs. The maintenance of high affinity MBCs was impaired in CDH17^-^ KO mice, and the differentiation of PCs from MBCs decreased; therefore, the replenishment of high affinity antibodies also decreased. LLPCs, which developed during the early phase, continued to produce low affinity antibodies for a long time; thus the amount of antibodies produced by LLPCs in WT and KO was the same because PCs do not express CDH17. Hence, after a long time post-antigen immunization, the high affinity antibody ratios in KO mice decreased and the total affinity antibody titer also decreased (slightly), even though the differences were not statistically significant.

Our data showed that the cell cycle turnover of CDH17^+^IgG1^+^ MBCs was faster than that of CDH17^-^ MBCs. Recent reports examining CDH17^+^ cancer cells reveal that cell cycle progression is slowed after CDH17 knockdown [18, 19]. The Wnt signaling pathway forms part of the cadherin signaling pathway [20], which regulates the expression of cyclin D1. Immunoprecipitation experiments show that CDH17 associates with β-catenin, although CDH17 has no catenin-binding domain in its intracellular region [21]. These findings suggest that CDH17 accelerates cell cycle progression in CDH17^+^ switched IgG1^+^ MBCs via the Wnt signaling pathway.

In humans, CDH17 is expressed in the fetal liver and gastrointestinal tract [22] but is downregulated in the adult liver and gastrointestinal tract; however, it is upregulated in gastric cancer, esophageal cancer, pancreatic cancer, and hepatocarcinoma [18, 23, 24]. CDH17 is also expressed on human B cell lines [25] and CD19^+^ peripheral blood lymphocytes [26], suggesting a role for CDH17 in human B cells. However, the exact role of CDH17 in human MBCs remains unclear.

It is thought that MBCs and memory T cells require a specific survival niche for long-term maintenance [27–30]. The survival niches for memory CD4^+^ T cells and LLPCs are well documented [31–33]. Memory CD4^+^ T cells and LLPCs are maintained by IL-7^+^ stromal cells and by CXCL12^+^ stromal cells, respectively, in the bone marrow. The number of maintained memory cells is limited by the available space within the survival niche. The methods used to create space for newly emerged memory cells within the survival niche have been reported [34]. However, it is still unclear whether stromal cells are required for MBC survival and, if so, what kind of stromal cells were required [27, 35, 36]. Here, we showed that a fraction of MAdCAM-1^+^ BEC express CDH17. According Kraal et al., MAdCAM-1^+^ vascular cells in the spleen are marginal sinus endothelial cells [14]. MBCs reside within the MZ in rodents and humans [37–39]. We previously showed that the expression of CDH17 is especially high in the MZ [3] (see also Fig. 6). Thus, we consider that the CDH17^+^ BECs identified in the present study comprise a fraction of the marginal sinus that offers an area suitable for CDH17-mediated homophilic-adhesion to MBC. Interestingly, recent studies show that endothelial cells within sinusoids comprise part of the stromal cell population that constitutes the hematopoietic stem cell niche [16, 27, 40]. However, we cannot exclude the possibility that other CDH17^+^ sessile-type cells, such as FRCs or other as-yet-unidentified cells, contribute to the MBC survival niche. The frequency of MAdCAM-1^+^CDH17^+^ BECs in the MZ sinus (MAdCAM-1^+^) was about 6% (Fig. 6E). We do not think this is too low to support MBCs because a recent report shows that FRCs comprise about 30% of total stromal (CD45^-^) cells and govern B cell homeostasis in mouse lymph nodes [41]. The memory-like IgG1^+^ B cells shown in Fig. 6C are CDH17^+^. Collectively, these data suggest that a fraction of MAdCAM-1^+^ BEC, which expresses CDH17, comprises the MBC survival niche and that MBCs are anchored within this niche by homotypic adhesion mediated by CDH17.

Recent reports on MBC localization in the mouse spleen identify the periphery of contractile GC as the region in which MBCs are maintained [28, 29, 39]. As for the splenic microarchitecture around the GC and MZ, the inner layer of MOMA-1^+^ metallophilic macrophages occasionally associates with the outer layer of GC in which CDH17 is also expressed. The difference between their experiments and ours is the timing of MBC examination: 8–25 weeks after the primary immunization in their case and after around 60 weeks in ours. The respective difference in the age of the MBCs would be reflected in the quality of the MBC population. In this context, it is noteworthy that we examined two populations of MBCs: CDH17^+^ and CDH17^-^. Our current hypothesis is that these two types of MBC are generated by asymmetric cell division (“self-renewal” and “differentiation-oriented”) on CDH17^+^ BEC, as observed for hematopoietic stem cells [42]. We speculate that the fate of CDH17^+^ and CDH17^-^ MBCs is as follows: whereas CDH17^+^ MBCs remain within the CDH17^+^ BEC niche and are maintained by homeostatic proliferation (which is presumably induced by catenin-mediated signaling via cell surface CDH17), CDH17^-^ MBCs may leave the niche and differentiate into PCs, which partly replenish long-term antigen-specific antibodies. The differentiation of CDH17^-^ MBCs into PCs requires antigen. We speculate that, after asymmetric cell division, CDH17^-^ MBCs leave the MBC niche and circulate throughout the body until they encounter an antigen. If CDH17^-^ MBCs do encounter an antigen, the cell is activated and differentiates into PCs to produce high affinity antibodies (if it does not encounter an antigen, the cell may die). It was reported that antigen-specific memory follicular T cells (Tfh) are in the draining regional lymph node [43]. We speculate that such cells may play a role in the activation of circulating CDH17^-^ MBC. It would also be possible that circulating CDH17^-^ MBCs are activated by polyclonal stimulation such as bystander T cell help and CpG DNA [44]. In KO mice, CDH17^-^ MBCs localize out of the MBC niche in the spleen by adhering to molecules other than CDH17; hence, the cells lack a homeostatic proliferation signal. [16][45, 46][6][21][11]In conclusion, we showed that CDH17 plays an important role in the long-term survival of antigen-specific MBCs, thereby maintaining the capability for rapid antibody production during a secondary immune response. We found that a fraction of MAdCAM-1^+^ BEC may represent a candidate constituent of the MBC survival niche. Further elucidation of the molecular mechanisms underlying the role of CDH17 in MBC maintenance may make it possible to establish improved vaccination protocols.

## Supporting Information

S1 Fig Some MBC marker molecules are differentially expressed on CDH17^+^ and CDH17^-^ MBCs. (A) Expression profiles of two chemokine receptors, three adhesion molecules, and two co-stimulatory molecules on IgG1^+^ MBCs (Lin^-^B220^+^IgD^-^IgM^-^CD38^+^IgG1^+^) were examined by flow cytometry. Numbers represent the percentage (%) of CDH17^+^ cells in the IgG1^+^ MBC gate. (B) The mean fluorescence intensity (MFI) of each marker expressed on IgG1^+^ MBCs are plotted on a bar graph (n = 6; *P≤0.05 (Mann-Whitney U-test)).
(TIF)

S2 Fig The percentage of MBC precursor cells is similar in WT and CDH17^-/-^ mice. The percentages of different splenic B cell populations are plotted on a bar graph (analyzed as described in the legend to Fig. 1F) (n = 2 (PCs); n = 8 (GC B); n = 6 (other)). *P≤0.05, **P≤0.01, ***P≤0.001 (Student’s t-test)).
(TIF)

S3 Fig The percentage of total IgG1^+^ MBCs is similar in WT and CDH17^-/-^ mice. (A) IgG1^+^ MBCs (Lin^-^B220^+^IgD^-^IgM^-^CD38^+^IgG1^+^) obtained from CDH17^-/-^ mice and their WT littermates at 52 weeks after primary immunization with NP-CGG in alum were analyzed by flow cytometry. Numbers represent the percentage (%) of the indicated cell populations in the respective parental gates (shown on top of the panels). The same experiments described in Fig. 4. (B) The percentages of IgG1^+^ MBCs are plotted on a bar graph. The y-axis shows the percentage of IgG1^+^ MBCs (Lin^-^B220^+^IgD^-^IgM^-^CD38^+^IgG1^+^) in the respective B220^+^ parental gate. The number of weeks post-immunization is shown for each bin.
(TIF)
