# Supplementary figures and images for: BILL-Cadherin/Cadherin-17 Contributes to the Survival of Memory B Cells
Source: PLoS One. 2015 Jan 22;10(1):e0117566. doi: 10.1371/journal.pone.0117566 (PMC4303427; doi:10.1371/journal.pone.0117566)

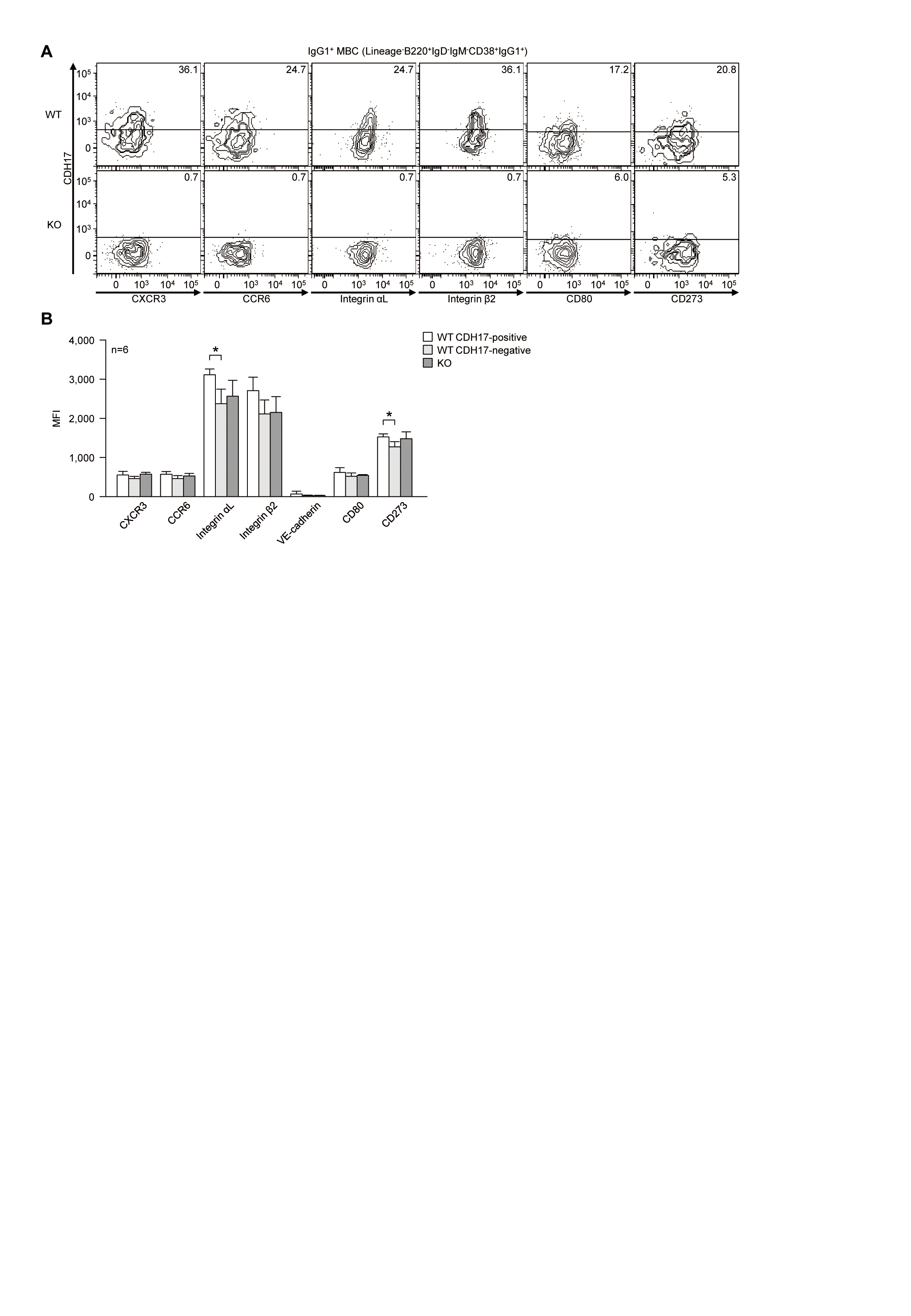

Supplement: S1 Fig — (A) Expression profiles of two chemokine receptors, three adhesion molecules, and two co-stimulatory molecules on IgG1+ MBCs (Lin-B220+IgD-IgM-CD38+IgG1+) were examined by flow cytometry. Numbers represent the percentage (%) of CDH17+ cells in the IgG1+ MBC gate. (B) The mean fluorescence intensity (MFI) of each marker expressed on IgG1+ MBCs are plotted on a bar graph (n = 6; *P≤0.05 (Mann-Whitney U-test)). (TIF) [file pone.0117566.s001.tif]

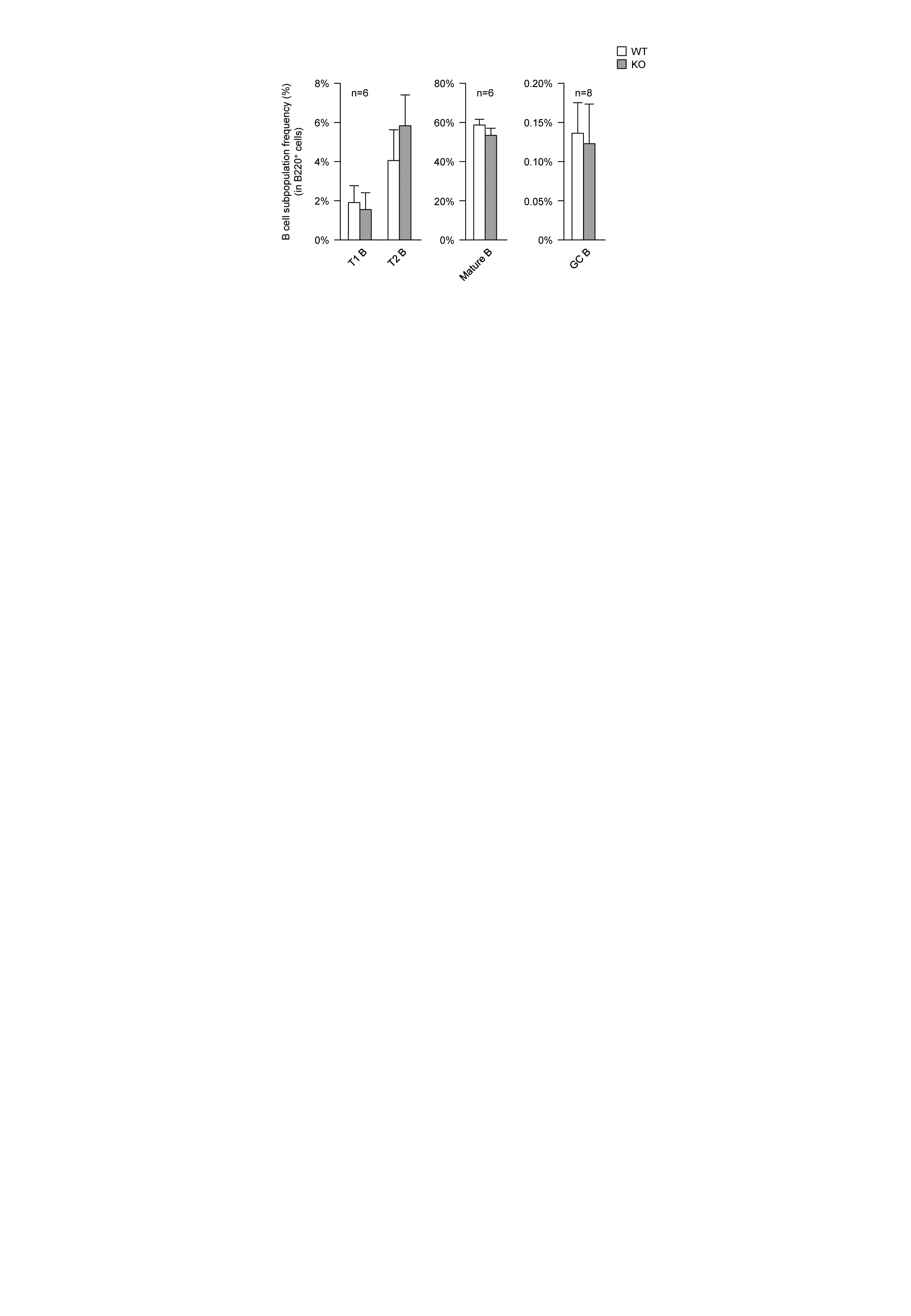

Supplement: S2 Fig — The percentages of different splenic B cell populations are plotted on a bar graph (analyzed as described in the legend to Fig. 1F) (n = 2 (PCs); n = 8 (GC B); n = 6 (other)). *P≤0.05, **P≤0.01, ***P≤0.001 (Student’s t-test)). (TIF) [file pone.0117566.s002.tif]

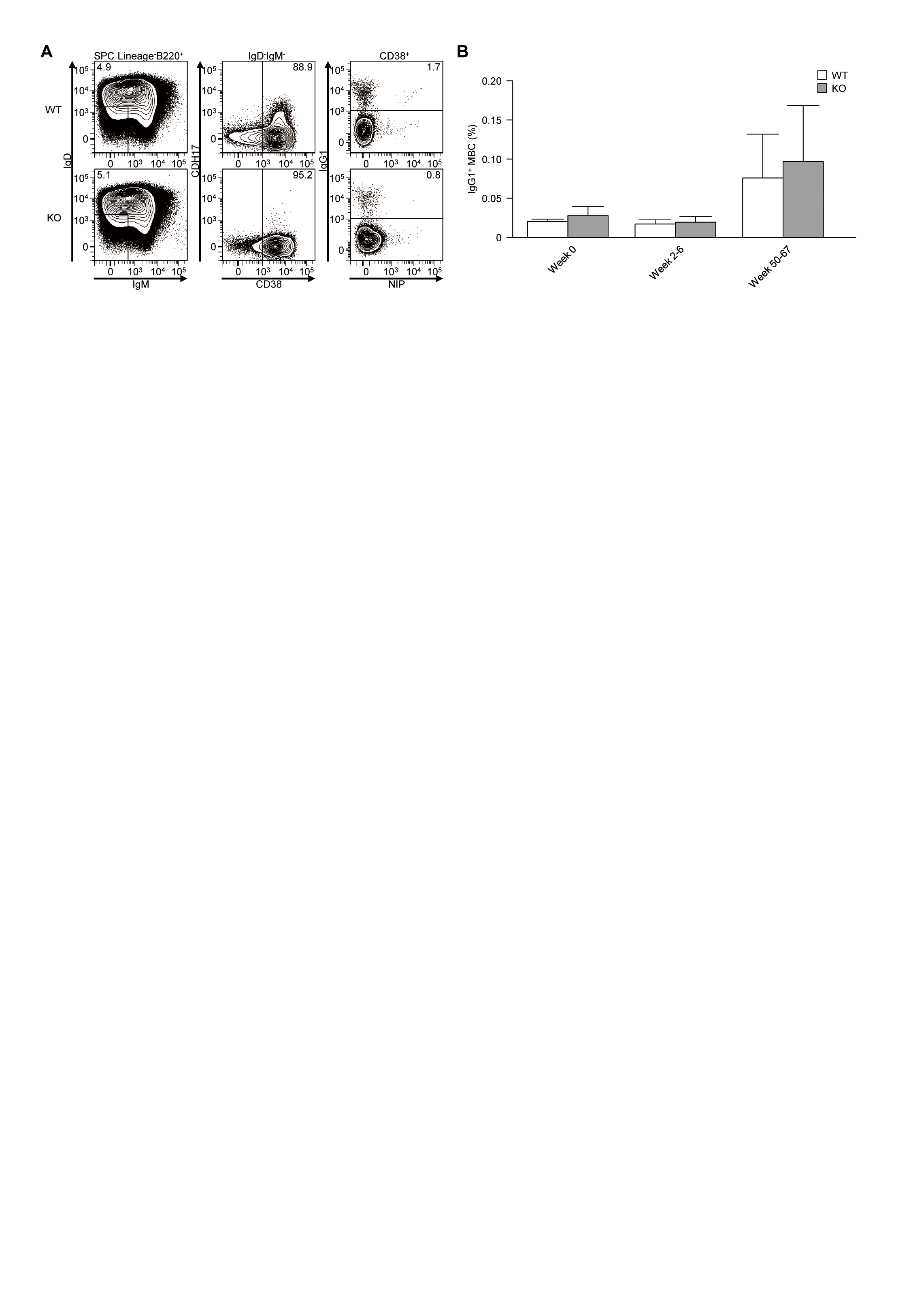

Supplement: S3 Fig — (A) IgG1+ MBCs (Lin-B220+IgD-IgM-CD38+IgG1+) obtained from CDH17-/- mice and their WT littermates at 52 weeks after primary immunization with NP-CGG in alum were analyzed by flow cytometry. Numbers represent the percentage (%) of the indicated cell populations in the respective parental gates (shown on top of the panels). The same experiments described in Fig. 4. (B) The percentages of IgG1+ MBCs are plotted on a bar graph. The y-axis shows the percentage of IgG1+ MBCs (Lin-B220+IgD-IgM-CD38+IgG1+) in the respective B220+ parental gate. The number of weeks post-immunization is shown for each bin. (TIF) [file pone.0117566.s003.tif]
